# Investigation of the Oxidation Behavior of Cr_20_Mn_17_Fe_18_Ta_23_W_22_ and Microdefects Evolution Induced by Hydrogen Ions before and after Oxidation

**DOI:** 10.3390/ma15051895

**Published:** 2022-03-03

**Authors:** Bao-Zhen Wu, Te Zhu, Xing-Zhong Cao, Zhao-Ming Yang, Kun Zhang, Fu-Jun Gou, Yuan Wang

**Affiliations:** 1 Key Laboratory of Radiation Physics and Technology, Ministry of Education, Institute of Nuclear Science and Technology, Sichuan University, Chengdu 610064, China; wbz42954@foxmail.com (B.-Z.W.); yangzhaoming0721@163.com (Z.-M.Y.); kzhang@scu.edu.cn (K.Z.); gfujun@scu.edu.cn (F.-J.G.); 2 Multi-Disciplinary Research Center, Institute of High Energy Physics, Chinese Academy of Sciences (CAS), Beijing 100049, China; zhute@ihep.ac.cn (T.Z.); caoxzh@ihep.ac.cn (X.-Z.C.)

**Keywords:** Cr_20_Mn_17_Fe_18_Ta_23_W_22_ refractory high-entropy alloy (RHEA), oxidized, defects, hydrogen ions irradiation

## Abstract

The oxidation behavior of body-centered cubic (bcc) structure Cr_20_Mn_17_Fe_18_Ta_23_W_22_ refractory high-entropy alloy (RHEA) and the microdefects induced by hydrogen ions before and after oxidation were investigated. The results revealed that compared with oxidizing Cr_20_Mn_17_Fe_18_Ta_23_W_22_ at 800 °C (6.7 °C/min) for 4 h (ST3, Ar:O_2_ = 3:1), the heating procedure of oxidizing Cr_20_Mn_17_Fe_18_Ta_23_W_22_ at 300 °C (6 °C/min) for 2 h and then increased to 800 °C (5 °C/min) for 4 h is more conducive to the production of oxides without spalling on the surface, i.e., HT1 (Ar:O_2_ = 1:1), HT2 (Ar:O_2_ = 2:1) and HT3 (Ar:O_2_ = 3:1) samples. The oxidation of Cr_20_Mn_17_Fe_18_Ta_23_W_22_ RHEA is mainly controlled by the diffusion of cations instead of affinities with O. Additionally, HT1 and HT3 samples irradiated with a fluence of 3.9 × 10^22^ cm^−2^ hydrogen ions (60 eV) were found to have a better hydrogen irradiation resistance than Cr_20_Mn_17_Fe_18_Ta_23_W_22_ RHEA. The microdefects in irradiated Cr_20_Mn_17_Fe_18_Ta_23_W_22_ mainly existed as hydrogen bubbles, hydrogen-vacancy (H-V) complexes and vacancy/vacancy clusters. The microdefects in irradiated HT3 were mainly vacancies and H-V complexes, while the microdefects in irradiated HT1 mainly existed as vacancies and vacancy clusters, as large amounts of hydrogen were consumed to react with oxides on the HT1 surface. The oxides on the surface of the HT3 sample were more stable than those on HT1 under hydrogen irradiation.

## 1. Introduction

Hydrogen isotopes (deuterium and tritium) are the primary fuel for nuclear fusion; however, the migration and retention of hydrogen in structural materials affect the fuel efficiency, which could lead to hydrogen embrittlement [1,2,3,4,5]. Therefore, research on preparing stable hydrogen permeation barriers to reduce hydrogen solubility and diffusivity has become an important issue in the study of nuclear materials [6,7,8,9]. To simulate the bombardment of the material in the fusion reactor by hydrogen isotopes plasma, hydrogen irradiation experiments have been conducted and investigated by researchers [10,11]. Hydrogen irradiation often produces lots of vacancy type defects, like vacancy and H-V complexes. These defects ultimately lead to irreversible plastic deformation of materials. Therefore, investigating the evolution of defects caused by hydrogen irradiation is crucial for the development of fusion reactor material. However, related research is rare to date. Lu et al. [12] observed monovacancies in FeMnNiCoCr HEA using positron annihilation spectroscopy (PAS) after irradiation with low doses of hydrogen. Ramachandran et al. [13] applied PAS to investigate the evolution of defects with temperature in RAFM steel irradiated by hydrogen and helium. The results revealed that H-vacancy complexes emerged after being irradiated with only hydrogen, and the S-parameter, which could reflect vacancy type defects, increased when hydrogen was released from H-V complexes at 373 K. However, after annealing at 673 K, the defects recovered completely.

High entropy alloys (HEAs), as new materials which are mainly composed of five or more elements with a single or two phases, have attracted lots of attention and are considered as a potential high-performance structural material due to their extraordinary properties, like high strength, hardness and irradiation resistance [14,15,16]. In the meantime, refractory high entropy alloys (RHEAs), a subcategory of of HEAs that contain refractory metal elements, have also been extensively studied in recent years due to their extraordinary mechanical properties at high temperature and the resistance to radiation damage. RHEAs also have been applied as structural materials in harsh environments like nuclear fusion reactors [17,18,19,20,21,22,23]. To promote their hydrogen permeation resistance properties, oxidizing RHEAs directly is a solution to increase the cohesiveness between the oxides and the RHEA structural material. However, the oxidation properties of RHEAs have not been fully addressed due to their complex composition [24]. Beyond their affinity with O, the element diffusion in alloy matrices and deuterogenic oxides also affect the oxidation process; nonetheless, related research has been minimal. For instance, compared with commercial stainless steels and Fe-Cr alloys, FeCoNiCrMn HEA displayed a sluggish diffusion behavior [25]; elements diffuse in FeCoNiCrMn HEA mainly through vacancies [26]. The sequence of diffusivities from fastest to slowest in CoCrFeMnNi composition was D_Mn_ > D_Cr_ ≈ D_Fe_ > D_Co_ ≈ D_Ni_ [27]. In addition, previous studies about the oxidation of Fe-Cr based alloys including Mn indicated that the diffusion rates of metal ions through chromium oxide are D_Mn_ > D_Fe_ > D_Ni_ > D_Cr_ [28]. The lattice diffusivity of Mn is greater than that of the other element (two orders of magnitude), while the values of Cr, Fe, and Ni are in the same scope in chromium oxide [29]. 

In this work, a BCC structure Cr_20_Mn_17_Fe_18_Ta_23_W_22_ RHEA coating was prepared by magnetron sputtering. The five elements are low neutron activation elements and satisfy the element selection requirements for a fusion reactor (Cr, Mn and Fe: no limit; Ta and W: several %) [30]. To form an effective hydrogen permeation barrier on a potential structural material, i.e., Cr_20_Mn_17_Fe_18_Ta_23_W_22_, selective oxidation was conducted, which is a simple preparation method. This material is suitable for use in tubular structures in fusion reactors. The oxidation behavior of Cr_20_Mn_17_Fe_18_Ta_23_W_22_ is discussed. Hydrogen ion irradiation tests were carried out at a plasma surface interaction (PSI) facility. The PSI facility is able to produce ions with low energy and high fluence. In addition, it can achieve both stress and temperature field corrosion, and simulate the reactor conditions more effectively than ordinary hydrogen (isotope) permeation tests. The evolution of microstructure defects in Cr_20_Mn_17_Fe_18_Ta_23_W_22_ before and after oxidation induced by hydrogen-ion irradiation was mainly investigated by the positron annihilation Doppler broadening technique. Low activation Cr_20_Mn_17_Fe_18_Ta_23_W_22_ with an oxide layer has the potential to prevent hydrogen irradiation and withstand high temperatures.

## 2. Materials and Methods

### 2.1. Materials

A Cr-Mn-Fe-Ta-W coating was deposited on Si (100) single crystal (Zhongnuo New Material Co., Beijing, China) substrates using a magnetron sputtering system (QX-600, CHENGDU QIXING VACUUM COATING TECHNOLOGY CO., LTD, Chengdu, China) with a power of 100 W for 2 h. Before the deposition, the Si substrates were ultrasonically washed in ethanol, acetone and deionized water, respectively. The Cr-Mn-Fe-Ta-W target (99.9% in purity) was obtained by mixed smelting 20% Cr, 20% Mn, 20% Fe, 20% Ta and 20% W (at.%). The target diameter was 50.8 mm and the base pressure was 4.0 × 10^−4^ Pa. The atom proportion of each element in the deposited Cr-Mn-Fe-Ta-W, as determined by EDS, was 20% Cr, 17% Mn, 18% Fe, 23% Ta and 22% W. Therefore, Cr_20_Mn_17_Fe_18_Ta_23_W_22_ RHEA was obtained after being normalized.

Then, Cr_20_Mn_17_Fe_18_Ta_23_W_22_ RHEA was oxidized in a tube furnace (BTF-1700C, BEST EQUIPMENT Co., Hefei, China). The first Cr_20_Mn_17_Fe_18_Ta_23_W_22_ sample was oxidized at 800 °C (6.7 °C/min) for 4 h; the ratio of argon (Ar) to oxygen (O_2_) was 3:1 (Ar:O_2_ = 90 sccm:30 sccm, i.e., 3:1) and the total oxidation time was 11 h (marked as ST3). Other samples were oxidized at 300 °C (6 °C/min) for 2 h, increasing to 800 °C (5 °C/min) for 4 h; the total oxidation time was 13.5 h. The ratios of argon to oxygen were 30 sccm:30 sccm (1:1), 60 sccm:30 sccm (2:1) and 90 sccm:30 sccm (3:1), respectively. These three oxidized samples were marked as HT1 (Ar:O_2_ = 1:1), HT2 (Ar:O_2_ = 2:1) and HT3 (Ar:O_2_ = 3:1). The entire process was aerated until the temperature dropped to 50 °C. The device was then turned off and the air outlet was closed.

### 2.2. Hydrogen Ions Irradiation Experiments

Hydrogen ion irradiation experiments were conducted at the plasma surface interaction (PSI) facility in Sichuan University. The base pressure was 5 × 10^−2^ Pa. Hydrogen plasma with an electron density of 1.69 × 10^19^ m^−3^ and electron temperature of 1.87 eV was obtained in a steady state. The magnetic field strength was 0.3 T and the hydrogen ion flux was 1.64 × 10^23^ m^−2^s^−1^. The hydrogen energy was 60 eV by adding a −60 V bias voltage to the target during irradiation. The exposure time was 40 min, and the corresponding fluence was 3.9 × 10^22^ cm^−2^. The temperature during irradiation was about 350 °C, which was measured by a thermocouple near the specimen.

### 2.3. Microstructure Characterization

Phase identification was conducted by Grazing incidence (2.0°) X-ray diffraction (GIXRD, Philips X Pert Pro MPD DY129, Cu Kα source, Philips, Amsterdam, Holland), and the surface morphology and cross-section microstructures were tested using a scanning electron microscope (SEM) equipped with EDS (JSM7500F, JEOL, Tokyo, Japan). Besides, to better identify the content of O element, X-ray Photoelectron Spectroscopy (XPS, AXIS Supra, Al Kα source, Kratos, Manchester, UK) analysis was conducted. The phase and element distribution were detected by a high-resolution transmission electron microscope (HRTEM, Libra200FE, Carl Zeiss AG, Oberkochen, Germany) with an energy dispersive spectrometer (EDS), and selected area electron-diffraction (SAED). TEM samples were prepared by mechanical polishing to a thickness of ~50 μm, followed by ion milling (Leica EM RES101, Leica, Weztlar, Germany) to no more than 200 nm.

The positron annihilation Doppler broadening spectroscopy (DBS) method, which can reflect vacancy type defects, was carried out on the slow positron beam device at the Institute of High Energy Physics (IHEP) [31]. The positron energy ranged from 0.18 keV to 20.18 keV. In the DBS spectra, the S parameter conveys the vacancy defect information of the sample, and the W parameter provides precipitation information from around the positron annihilation sites [32,33]. According to the two-state capture model of positrons captured by defects, S and W can be expressed by the following relationship:

(1) $$S=\left(1-f\right)S_{b}+fS_{d},$$

(2) $$W=\left(1-f\right)W_{b}+fW_{d}$$

According to Equations (1) and (2), $S=R\times \left(W-W_{b}\right)+S_{b}$ could be obtained. f represents the share of positron annihilation in the defect, and $S_{b}\left(W_{b}\right)$ and $S_{d}\left(W_{d}\right)$ refer to the specific S and W value of the free state of the positive electron and the bound state of the defect, respectively. The slope of S−W curve $R=(S_{d}-S_{b})/\left(W_{d}-W_{b}\right)\;$is only related to the defect type. Therefore, the change of defect type can be judged according to the change of slope of the S−W curve.

## 3. Results and Discussion

### 3.1. Microstructures of the Deposited Cr_20_Mn_17_Fe_18_Ta_23_W_22_ RHEA

**Figure 1:**
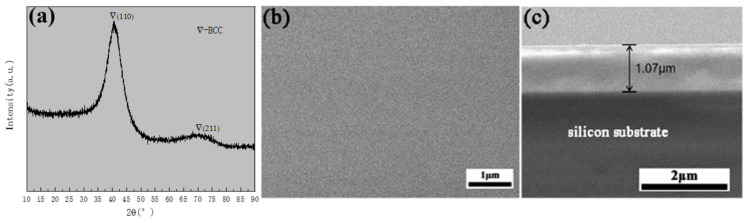
As-deposited Cr_20_Mn_17_Fe_18_Ta_23_W_22_ RHEA: (**a**) The GIXRD pattern; (**b**) SEM surface image; (**c**) Cross-section SEM image.

**Figure 2:**
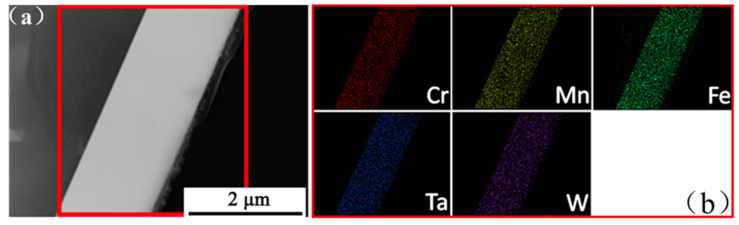
The as-deposited Cr_20_Mn_17_Fe_18_Ta_23_W_22_: (**a**) The cross-sectional HAADF image; (**b**) EDS-mapping images.

The GIXRD pattern of Cr_20_Mn_17_Fe_18_Ta_23_W_22_ RHEA revealed that the structure of the as-deposited Cr_20_Mn_17_Fe_18_Ta_23_W_22_ was a BCC phase (Figure 1a). Additionally, the BCC peak had a significant broadening as the grain of Cr_20_Mn_17_Fe_18_Ta_23_W_22_ RHEA was nanocrystalline and the grain size was ~9 nm. Therefore, it could be deduced that there were lots of grain boundaries due to the small nanocrystals, and that these boundaries could provide channels for element diffusion and promote the oxidation process. The surface morphology of the as-deposited specimen was smooth and intact, and the thickness was ~1.07 μm (Figure 1b,c). Additionally, it was found that the five elements in Cr_20_Mn_17_Fe_18_Ta_23_W_22_ were evenly distributed according to the EDS mapping results shown in Figure 2.

### 3.2. Oxidation of Cr_20_Mn_17_Fe_18_Ta_23_W_22_ RHEA

**Figure 3:**
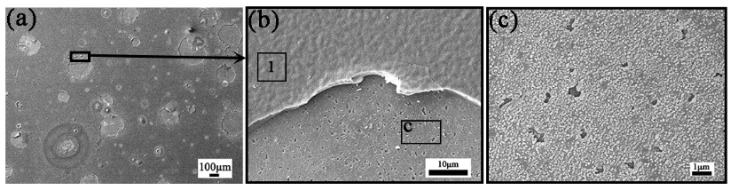
(**a**) The SEM surface morphology image of the ST3 sample (i.e., Cr_20_Mn_17_Fe_18_Ta_23_W_22_ coating after oxidization at 800 °C (6.7 °C/min) for 4 h (Ar:O_2_ = 3:1)); (**b**) The enlarged SEM surface morphology image of the region marked with black box in (**a**); (**c**) The enlarged SEM surface morphology image of the region marked with letter c and black box in (**b**).

Figure 3 shows SEM images of Cr_20_Mn_17_Fe_18_Ta_23_W_22_ after oxidization at 800 °C (6.7 °C/min) for 4 h (ST3). Lots of spallation emerged on the surface as shown in Figure 3a,b. Besides, according to Figure 3c, it can be found that there are lots of pores in the c region of Figure 3b. To investigate the element content of the oxide layer on the surface, EDS and XPS were conducted, as shown in Table 1. The depth of the EDS test reached ~1μm of the samples, but the oxygen content could not be measured accurately by EDS due to the light atomic weight of the element, so the relative content of each metal element was obtained by subtracting the O content and then normalizing. Additionally, to investigate the O content, an XPS test was conducted. However, the XPS test could only reach a depth of several nanometers, so the results only reflected the outermost oxide layers of the samples, and one or several kinds of oxides could be identified. Combined with the EDS and XPS results, it could be deduced that lots of O was distributed on the surface, and that the oxides mainly consisted of Mn, Fe and Cr oxides, with Mn oxides being distributed on the outermost layer.

**Figure 4:**
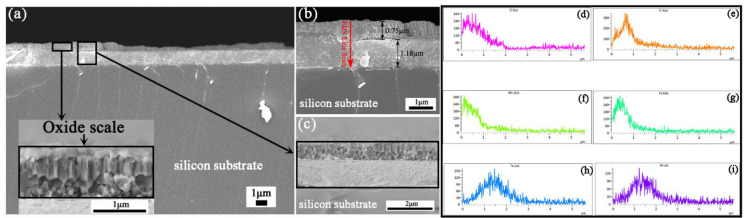
ST3 specimen: (**a**–**c**) Cross-section images; (**d**–**i**) EDS line scan of cross-section of the coating after being oxidized. The line scan followed the direction of the red arrow in (**b**).

The cross-sectional images in Figure 4a–c shows that the thickness of the oxidized coating increased to ~1.93 μm, and the spallation was mainly composed of the oxidation scale. Besides, most oxidation scales presented as irregular polygons, especially columnar polygons. Additionally, according to the EDS line scan in Figure 4d–i, most of the Mn was distributed on the outer surface, followed by Fe and Cr, while Ta and W stayed at the bottom (Mn-Fe-Cr rich region: ~0.75 μm, Ta-W rich region: ~1.18 μm). The Pauling electronegativity of each alloy is shown in Table 2. Generally, metallic elements with lower electronegativity values tend to be oxidized first; as such, the order of oxidation would be Ta, Mn, Cr, Fe and W. However, our results were not completely consistent with this trend. Ta and W were distributed as expected, i.e., the bottom layer, while Mn, Fe, Cr and O were distributed on the surface. This is ascribed to Ta and W, as the refractory metallic elements with a high melting point are not easy to spread. Meanwhile, Mn, Fe and Cr diffuse more rapidly [25], and the related oxides form as a barrier, further hindering the oxidation of Ta and W. After more and more oxide of Mn, Fe and Cr formed, the volume expansion of each oxide was different, which inevitably led to the generation of internal stress in a tension or compression way, ultimately leading to spallation.

Based on the analysis above, it was found that the oxidation process of Cr_20_Mn_17_Fe_18_Ta_23_W_22_ RHEA was mainly controlled by the outward diffusion of metal cations in the alloy and oxides. Additionally, numerous pores formed in the Ta-W layer adjacent to the Mn-Fe-Cr oxide scale (Figure 3c). This was attributed to the vacancies generated by the rapid diffusion of Mn, Fe and Cr ions, combining to form pores [34,35]. Thus Mn, Fe and Cr were depleted in the Ta-W layer. Moreover, pores and cracks created the main channels for element diffusion and accelerated the oxidation process [36].

**Figure 5:**
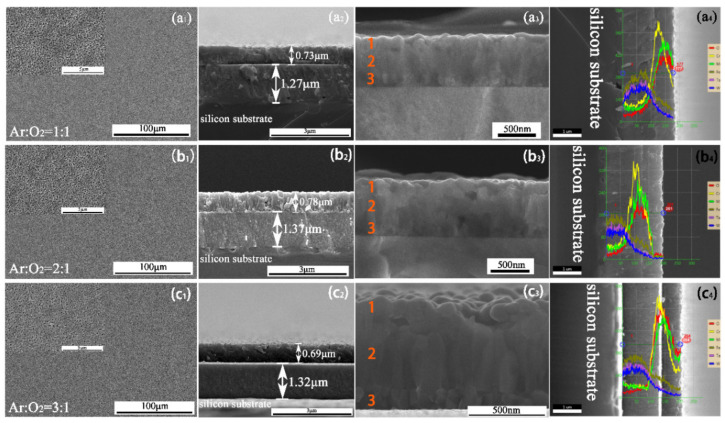
The morphology and element distribution of S_1_, HT2 and HT3: (**a_1_**–**c_1_**) SEM surface images; (**a_2_**,**a_3_**), (**b_2_**,**b_3_**), (**c_2_**,**c_3_**) Cross-section images; (**a_4_**–**c_4_**) EDS line scan images.

To improve the spalling phenomena, the heating process was changed. Figure 5 exhibits the surface and cross-section topographies of specimens oxidized at 300 °C for 2 h, and then increased to 800 °C for 4 h. The ratios of argon to oxygen were 3:1, 2:1, 1:1 in the HT1, HT2 and HT3 samples, respectively. No obvious spalling or cracks on the oxide scale were observed, but lots of tiny voids were distributed on the outer surface according to the high magnification shown in Figure 5(a_1_–c_1_), which could facilitate the entry of oxygen. According to Figure 5(a_2_–c_2_), the thickness of the three coatings, i.e., those on HT1, HT2 and HT3, were ~2 μm (oxidation layer: ~0.73 μm, bottom layer: ~1.27 μm), 2.15 μm (oxidation layer: ~0.78 μm, bottom layer: ~1.37 μm) and 2.01 μm (oxidation layer: ~0.69 μm, bottom layer ~1.32 μm), respectively (Figure 5(a_2_–c_2_)). The oxide scale present in Figure 5(a_3_–c_3_) may be divided into three regions (marked as 1, 2 and 3, respectively). The superficial oxide layer (1) presented a granular structure, the middle layer (2) was columnar, and the bottom oxide layer (3), which connected the Fe-Ta-W layer, presented as polygonal prisms.

To investigate the distribution of elements, EDS line scans were conducted. Figure 5(a_4_–c_4_) reveals that most Mn was distributed on the outer surface, followed by Cr, while most O was distributed in the Mn-Cr-rich layer. The distribution trends of Fe, Ta and W were consistent, i.e., these elements stayed in the bottom layer. Table 3 lists the contents of each element on the surface of HT1, HT2 and HT3. The analysis method was similar to that used in Table 1. Combined with the EDS and XPS results, it can be deduced that lots of O was distributed on the surfaces of the HT1, HT2 and HT3 samples. The oxides mainly consisted of Mn and Cr oxides, although Mn oxides were also distributed in the outermost layer. 

**Figure 6:**
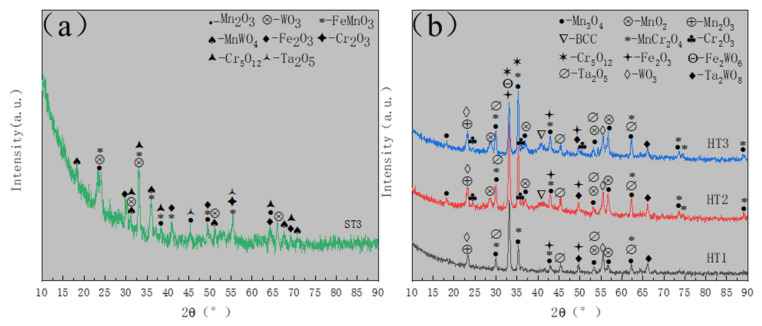
(**a**) The GIXRD pattern of ST3, i.e., Cr_20_Mn_17_Fe_18_Ta_23_W_22_ RHEA oxidized at 800 °C (6.7 °C/min) for 4 h (Ar:O_2_ = 3:1); (**b**) The GIXRD results of HT1, HT2 and HT3, i.e., Cr_20_Mn_17_Fe_18_Ta_23_W_22_ RHEA oxidized at 300 °C (6 °C/min) for 2 h and then increased to 800 °C (5 °C/min) for 4 h; the ratios of argon to oxygen were 3:1, 2:1 and 1:1, respectively.

The GIXRD pattern of the ST3 specimen is shown in Figure 6a. Combined with the EDS line scan results (Figure 4d–i), it can be deduced that the oxides mainly comprised of Mn_2_O_3_, FeMnO_3_, Fe_2_O_3_, Cr_2_O_3_ and Cr_5_O_12_.Few WO_3_, MnWO_4_ and Ta_2_O_5_. Figure 6b shows the GIXRD patterns of the other three samples, i.e., HT1, HT2 and HT3 respectively. Combining with EDS line scan results (Figure 5(a_4_–c_4_)), the oxides of the three samples were shown to mainly contain MnO_2_, Mn_3_O_4_, Mn_2_O_3_, MnCr_2_O_4_, Cr_2_O_3_ and Cr_5_O_12_, and small amounts of Fe_2_O_3_, Fe_2_WO_6_, Ta_2_O_5_, WO_3_ and Ta_2_WO_8_. Compared with ST3 and HT1, the BCC phase was detected in HT2 and HT3, illustrating that the degrees of oxidation were not as strong as those of ST3 and HT1. The peak intensity of the BCC phase in HT3 surpassed that of HT2. Additionally, Cr_2_O_3_ in HT3, as the predecessor of Cr_5_O_12_, emerged more than HT2. This indicated that the degree of oxidation decreased as the ratio of argon to oxygen increased.

Therefore, it was found that under the two different heating procedures, different oxidation degrees and oxide products were obtained, and the distribution of Fe was different. These two heating procedures reflect two different oxidation rates of Cr_20_Mn_17_Fe_18_-Ta_23_W_22_ RHEA, as determined by calculating the unit time variation of film thickness before and after oxidation. Representative samples were ST3 and HT3, which were both under the same gas environment, i.e., Ar:O_2_ = 3:1. To compare the effects of different oxidation rates on oxidation behavior, we further studied microstructural differences in ST3 and HT3 more precisely through TEM.

**Figure 7:**
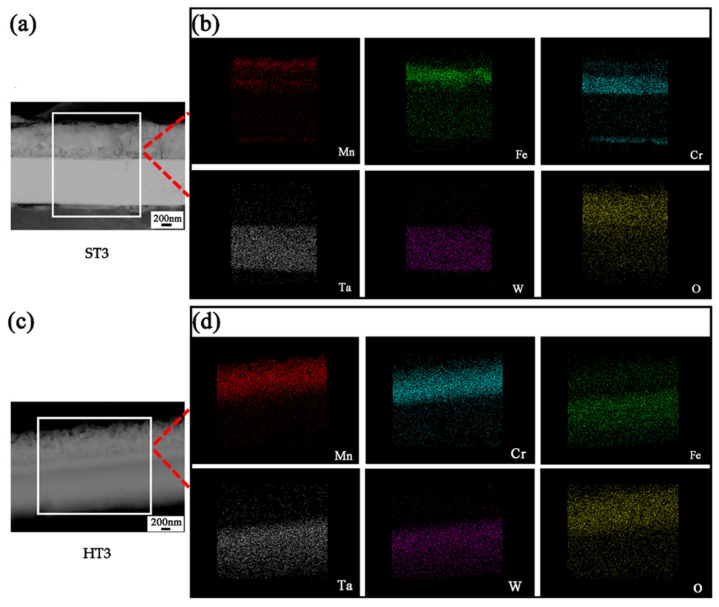
(**a**) The cross-sectional HAADF image of ST3 sample; (**b**) The EDS–mapping image of ST3 sample; (**c**) The cross-sectional HAADF image of HT3 sample; (**d**) The EDS–mapping image of HT3 sample.

**Figure 8:**
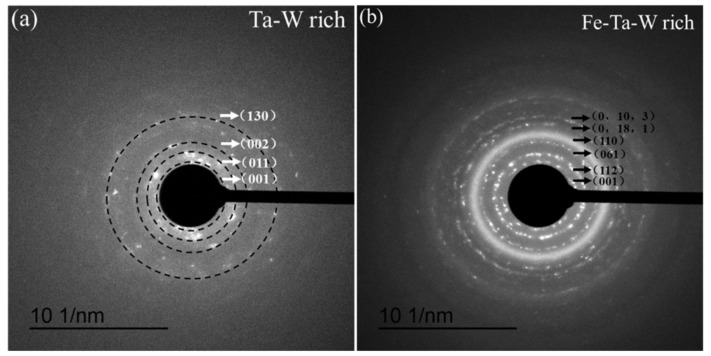
(**a**) The selected area electron diffraction (SAED) pattern of the Ta-W rich layer in the ST3 sample; (**b**) SAED patterns of the Fe-Ta-W rich layer in the HT3 sample.

Figure 7a,c is the cross-sectional HAADF images of ST3 and HT3 samples, while Figure 7b,d presents the EDS-mapping images of ST3 and HT3 samples. The results of EDS-mapping about the distribution of each element in ST3 sample are consistent with the EDS line scan results in Figure 4d–i. Most O was distributed in the Mn-Fe-Cr layer, although a little was present in the Ta-W layer. The SAED pattern of the Ta-W rich layer in ST3 is shown in Figure 8a. This demonstrates that WO_3_ ((001) and (011)) and MnWO_4_ ((002) and (130)) existed in the Ta-W rich layer, which was consistent with the XRD result (Figure 6a).

Therefore, it can be deduced that during the oxidation process of ST3, the diffusion of Mn was fastest in Cr_20_Mn_17_Fe_18_Ta_23_W_22_ RHEA, followed by Cr and Fe, due to D_Mn_ > D_Cr_ ≈ D_Fe_ [25], as such, Mn_x_O_y_ formed at first. As the inner diffusion of oxygen and the Pauling electronegativity of Cr are lower than that of Fe (Table 2), Cr_x_O_y_ also formed. The diffusion rates of metal ions through chromium oxide are D_Mn_ > D_Fe_ > D_Cr_ [37,38]. Therefore, Fe would have diffused through Cr_x_O_y_ and formed Fe_x_O_y_. A little Ta_x_O_y_ and W_x_O_y_ formed due to the inner diffusion of oxygen, and a small amount of MnWO_4_ formed by MnO reacting with WO_3_.

In HT3, most O clustered in the Mn-Cr-rich zone, although a little was present in the Fe-Ta-W layer (Figure 7d). WO_3_ (001) and (112), Fe_2_WO_6_ (061), Ta_2_O_5_ (0,18,1) and Ta_2_WO_8_ (0,10,3) existed in the Fe-Ta-W rich layer of HT3, according to the SAED result presented in Figure 8b. BCC (110) was also detected in the Fe-Ta-W rich area. This is consistent with EDS and XRD results shown above.

Combined with the GIXRD, EDS line scans, EDS mapping and the SAED and HRTEM results, it can be concluded that for HT3, Mn diffused firstly in Cr_20_Mn_17_Fe_18_Ta_23_W_22_ RHEA and formed Mn_x_O_y_, before Cr_x_O_y_ was formed. This process is the same as that observed in ST3. However, the heating process remained at 300 °C and the oxidation rate slowed down, which may have facilitate the rapid formation of MnCr_2_O_4_. A MnCr_2_O_4_ spinel could act as a protective coating to prevent the diffusion of Fe^3+^ or Fe^2+^ and restrict the formation of Fe oxide.

**Figure 9:**
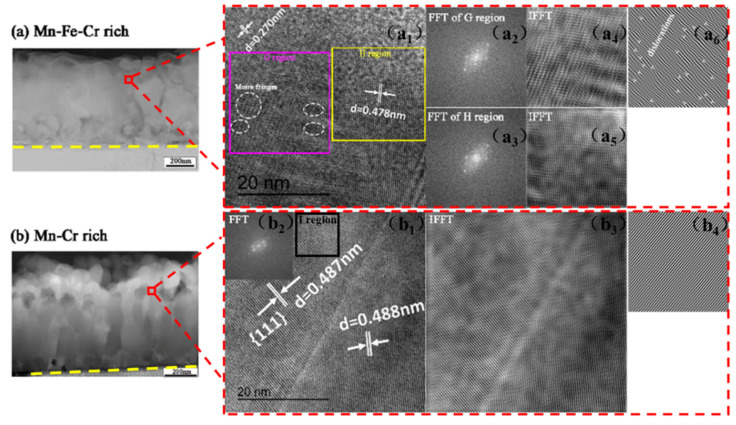
(**a**) HAADF images of the oxide layer of ST3 sample; (**a_1_**) HRTEM images of a selected region in (**a**); (**a_2_**,**a_3_**) Fast Fourier transform (FFT) images of G and H regions in (**a_1_**); (**a_4_**,**a_5_**) Inverse Fast Fourier transform (IFFT) images of G and H regions (filter); (**a_6_**) IFFT image of G region; (**b**) HAADF image of oxide layer of HT3 sample; (**b_1_**) HRTEM image of selected region in (**b**); (**b_2_**) FFT image of (**b_1_**,**b_3_**); IFFT image of (**b_1_**) (filter); (**b_4_**) IFFT image of I region in (**b_1_**).

Figure 9a,b shows HAADF and HRTEM images of the oxide scales of ST3 and HT3. As shown in Figure 9(a_2_), a double lattice appeared, like a “satellite spot”, and lots of moire fringes distributed in the G region. Furthermore, plenty of edge dislocations and lattice distortion existed in the G region after oxidation, as shown in Figure 9(a_6_), while in the H region, the grain spacing was clear, and no moire fringes and little lattice distortion were observed. To a certain extent, this revealed that after some oxidation scale had peeled off in the form of flakes (see Figure 3), stress was released and lots of twisted lattice fringes and edge dislocations stayed. It can be inferred that during the oxidation process, the growth stress and the relatively high thermal stress resulted in a high density of dislocation close to the material surface. In turn, these defects accelerated oxidation [39]. In addition, we report that the activation energy of the diffusion element along the dislocation decreased to 1/2 that of the body diffusion [40]. This also explains the higher oxidation rates observed with ST3 compared to HT1, HT2 and HT3.

According to Figure 9(b_1_,b_2_), which present HRTEM and FFT images of the selected region in Figure 9b, the grain was polycrystal. Combined with the XRD results (Figure 6), it could be deduced that the grain comprised MnCr_2_O_4_ ({111}) polycrystals. Compared with ST3 sample, there were no moire fringes, edge dislocations or lattice distortion in the polycrystals according to the IFFT (b_3_) and IFFT (b_4_) filters, and the dislocation density was relatively low.

### 3.3. Oxidized Cr_20_Mn_17_Fe_18_Ta_23_W_22_ Irradiated with Hydrogen Ions (60 eV)

**Figure 10:**
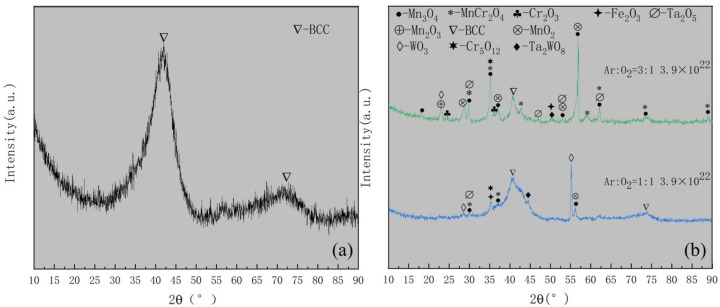
The GIXRD patterns the specimens irradiated with fluence of 3.9 × 10^22^ cm^−2^ hydrogen ions: (**a**) Cr_20_Mn_17_Fe_18_Ta_23_W_22_; (**b**) HT1 (Ar: O_2_ = 1:1) and HT3 (Ar:O_2_ = 3:1) specimens.

Based on the analysis above, HT1 (Ar:O_2_ = 1:1) and HT3 (Ar:O_2_ = 3:1) were chosen to study the hydrogen permeation resistance properties after the oxidation of Cr_20_Mn_17_Fe_18_Ta_23_W_22_; these represent two extreme oxidation degrees under the same heating procedure. Figure 10 shows the GIXRD patterns of Cr_20_Mn_17_Fe_18_Ta_23_W_22_, HT1 and HT3 specimens irradiated with a fluence of 3.9 × 10^22^ cm^−2^ hydrogen ions (60 eV). Compared with Figure 1a, it may be seen that the phase of Cr_20_Mn_17_Fe_18_Ta_23_W_22_ was stable after irradiation with hydrogen ions (Figure 10a). However, comparing the GIXRD results of the original HT1 and HT3 samples in Figure 6b, it can be found that HT1 underwent a remarkable change after irradiation (see Figure 10b), i.e., Mn_2_O_3_ disappeared, a BCC peak appeared, and some diffraction peaks of other oxides also largely disappeared, indicating that HT1 had undergone hydrogen reduction to a large extent. Zhang et al. [41] reported that high entropy alloys exhibit stronger activation than pure metals under hydrogen exposure, and that the oxides on the surface of HEA are easily reduced. This is consistent with our results. However, for HT3 irradiated with hydrogen ions, no oxide disappeared, but rather, only the diffraction peak intensities of some oxides changed, while the diffraction peak of BCC phase became slightly stronger (Figure 10b), indicating that there was a certain degree of hydrogen reduction, albeit not as significant as that observed with HT1. This may have been due to the presence of Cr_2_O_3_ in HT3, as Cr_2_O_3_ has good hydrogen permeation resistance properties [42]. Therefore, it can be considered that the oxides on the surface of HT3 were more stable and had better hydrogen permeation resistance than those on the surface of HT1.

**Figure 11:**
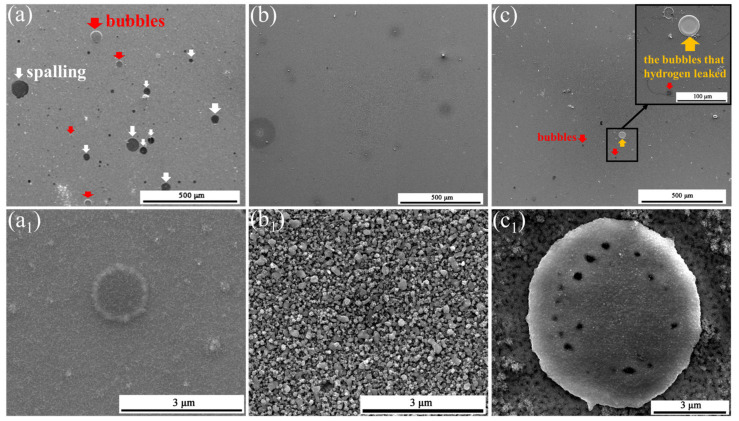
Surface SEM images of specimens irradiated with fluence of 3.9 × 10^22^ cm^−2^ hydrogen ions: (**a**,**a_1_**) Cr_20_Mn_17_Fe_18_Ta_23_W_22_; (**b**,**b_1_**) HT1 (Ar:O_2_ = 1:1) and (**c**,**c_1_**) HT3 (Ar:O_2_ = 3:1).

Figure 11 presents surface SEM images of Cr_20_Mn_17_Fe_18_Ta_23_W_22_, HT1 and HT3 irradiated with hydrogen ions. Compared with the surface SEM images of the original samples (Figure 3), it can be seen that lots of bubbles and spalling appeared on the surface of Cr_20_Mn_17_Fe_18_Ta_23_W_22_ after irradiation, and a shallow layer of fuzz was observed, as shown in Figure 11(a_1_). This may have been caused by the formation and breakup of tiny hydrogen bubbles. According to Figure 11b, no bubbles or spalling were observed on irradiated HT1, while the morphology of the particles on the surface changed compared with that in Figure 5(a_1_). This was consistent with the GIXRD result presented in Figure 10b, i.e., most oxides on the HT1 samples were reduced by hydrogen. Figure 11(c,c_1_) shows surface images of irradiated HT3. It can be seen that few bubbles emerged on HT3 after irradiation. Comparing Figure 11(c_1_) with Figure 5(c_1_) and Figure 11(b_1_), it can be seen that the morphology of the particles on the surface of irradiated HT3 did not change much; this was also consistent with the GIXRD result, in which few oxides on irradiated HT3 were shown to be reduced.

To investigate the evolution of vacancy-type defects of Cr_20_Mn_17_Fe_18_Ta_23_W_22_, HT1 and HT3 specimens before and after hydrogen ion irradiation, slow positron annihilation spectra were recorded. Generally, the higher the positron energy, the deeper it entered into the sample. The vacancy/vacancy cluster and H-V complex/hydrogen bubbles could be detected by PAS. The H-V complex formed due to some vacancies that would be trapped by hydrogen during hydrogen bombardment, and the H-V complex could also absorb more hydrogen and form hydrogen bubbles.

**Figure 12:**
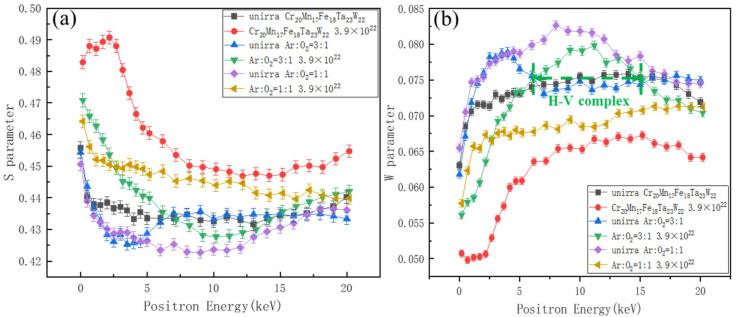
(**a**,**b**) S and W parameters as a function of the positron energy for Cr_20_Mn_17_Fe_18_Ta_23_W_22_; HT1 and HT3 specimens before and after H^+^ irradiation, respectively.

Figure 12a,b present the S and W parameters as a function of positron energy for the specimens before and after irradiation with hydrogen ions, respectively. It can be seen that the S value of Cr_20_Mn_17_Fe_18_Ta_23_W_22_ after irradiation was the largest (see Figure 12a), indicating the presence of vacancy defects in Cr_20_Mn_17_Fe_18_Ta_23_W_22_ after irradiation. According to the surface SEM images of HT1 and HT3 shown in Figure 5, the oxides on the surface were sparse and contained a large number of open volume defects; therefore, the S values near the surface of HT1 and HT3 were the highest, as shown in Figure 12a. Additionally, it can be seen that the S value of the HT1 and HT3 decreased to a relatively stable value and then began to increase; this was mainly due to the diffusion of elements to the surface during selective oxidation, and the presence of lots of vacancy defects in the Fe-Ta-W layer.

Additionally, it was found that for the HT1 specimen, the S value after irradiation was larger than that of primary HT1, indicating that a large number of vacancy type defects were produced. Additionally, most oxides in HT1 were reduced; therefore, it can be deduced that the hydrogen ions in HT1 were consumed, and the defects mainly existed as vacancies or vacancy clusters in HT1 after irradiation. However, the S value of HT3 after irradiation was smaller than that of primary HT3 when the positron energy ranged from 7.18 keV to 14.18 keV. This may have been due to the formation of H-V complexes, which could result in a decrease of S; the higher the proportion of H in the H-V complex, the lower the value of S [43]. This was confirmed by comparing S with the evolution of W, as shown in Figure 12a,b.

**Figure 13:**
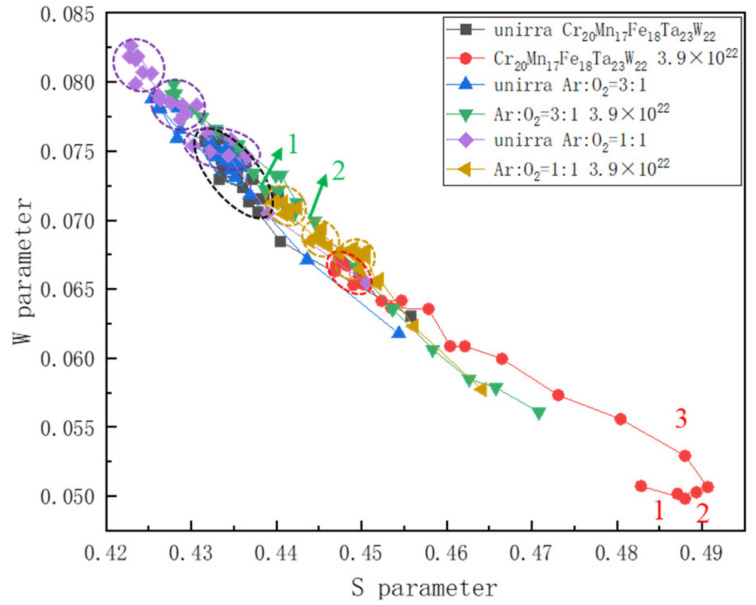
W—parameter as a function of S—parameter for Cr_20_Mn_17_Fe_18_Ta_23_W_22_, HT1 and HT3 specimens before and after H^+^ irradiation. The numbers represent the slope deviations.

Figure 13 shows the S−W curve of Cr_20_Mn_17_Fe_18_Ta_23_W_22_, HT1 and HT3 specimens before and after hydrogen ion irradiation. The S−W curve presenting as a linear function indicated that only one defect type existed [44]. It was found that the (S, W) points of the specimens before irradiation were mostly distributed in regions with smaller S and larger W values, and that there were some (S, W) aggregation regions which represented small vacancy clusters (marked with black, blue and purple circles, respectively). However, the (S, W) points of the irradiated samples were mostly distributed in the region with larger S and smaller W values, indicating the generation of a certain number of vacancy type defects after irradiation.

According to Figure 13, the S−W curve slope of Cr_20_Mn_17_Fe_18_Ta_23_W_22_ after irradiation had three different degrees of deviation (marked with 1,2 and 3). This, combined with SEM, S−E and W−E plot results, it could be concluded that the microdefects mainly existed as hydrogen bubbles, H-V complexes and vacancy/vacancy clusters (marked with red circles). The S−W curve slope of HT1 after irradiation basically presented a straight line with some $\left(S,W\right)$ point aggregations (marked with yellow circles). Therefore, combined with the analysis of GIXRD and S−E plot results, it can be concluded that the defects were mainly vacancy (V) and vacancy clusters. However, for the HT3 after irradiation, the S−W curve of the irradiated samples deviated to two different degrees (marked 1 and 2), suggesting that two different types of defects emerged after irradiation. Combined with the analysis above, it can be concluded that the microdefects in HT3 after irradiation mainly existed as V and H-V complexes.

## 4. Conclusions

In this work, a Cr_20_Mn_17_Fe_18_Ta_23_W_22_ RHEA coating was fabricated via magnetron sputtering. The oxidation mechanism of Cr_20_Mn_17_Fe_18_Ta_23_W_22_ RHEA and the evolution of defects induced by hydrogen ion irradiation before and after oxidation were studied. The following conclusions can be drawn:

(1) The oxidation behavior of Cr_20_Mn_17_Fe_18_Ta_23_W_22_ RHEA is mainly controlled by the diffusion of cations instead of affinities with O. For the sample oxidized at 800 °C (6.7 °C/min) for 4 h (ST3, Ar:O_2_ = 3:1), the oxide scale with plenty of spallation and crack; on a micro-level, twisted lattice fringes and edge dislocations remained. These defects, formed during oxidation, facilitated the diffusion of metal cations and oxygen, and accelerated oxidation. However, after slowing the oxidation rates, i.e., oxidizing Cr_20_Mn_17_Fe_18_Ta_23_W_22_ at 300 °C (6 °C/min) for 2 h and then increasing the temperature to 800 °C (5 °C/min) for 4 h (HT1 (Ar:O_2_ = 1:1), HT2 (Ar:O_2_ = 2:1) and HT3 (Ar:O_2_ = 3:1) samples), the surface spalling improved, and no obvious twisted lattice fringes or edge dislocations were observed.

(2) The distributions of Fe in oxidized samples under two different heating procedures were different. For ST3, Fe^3+^ or Fe^2+^ diffused through Cr_x_O_y_, formed Fe_2_O_3_ and FeMnO_3_; therefore, the oxide scale was Mn-Fe-Cr oxide. However, for HT1, HT2 and HT3, compared with ST3, the heating process stayed at 300 °C and the oxidation rate slowed. Under these conditions, MnCr_2_O_4_ formed relatively rapidly and hindered the diffusion of Fe^3+^ or Fe^2+^, and as such, the formation of Fe oxide was restricted. Therefore, the oxide scale was Mn-Cr oxide, while Fe-Ta-W stayed at the bottom.

(3) Compared with Cr_20_Mn_17_Fe_18_Ta_23_W_22_, the oxidized Cr_20_Mn_17_Fe_18_Ta_23_W_22_, i.e., HT1 (Ar:O_2_ = 1:1) and HT3 (Ar:O_2_ = 3:1), demonstrated better hydrogen irradiation resistance. Microdefects in Cr_20_Mn_17_Fe_18_Ta_23_W_22_ after irradiation mainly existed as hydrogen bubbles, H-V complexed and vacancy/vacancy clusters. The microdefects in HT1 after irradiation mainly existed as vacancies and vacancy clusters, as large amount of hydrogen were consumed to react with oxides on the HT1 surface, while the microdefects in HT3 after irradiation were mainly vacancies and H-V complexes. Finally, the oxides on the HT3 surface were more stable than those of HT1.

## Figures and Tables

**Table 1:** Content of each element of Region 1 in Figure 3 by EDS and XPS results for the HT3 sample surface.

Elements	O	Cr	Mn	Fe	Ta	W
	at.%					
EDS (Region 1)	25.69	21.83	23.84	25.47	1.54	1.63
	-	29.38	32.08	34.28	2.07	2.19
XPS	65.33	5.06	18.31	9.56	0.25	0.47

**Table 2:** Pauling electronegativity of each element in Cr_20_Mn_17_Fe_18_Ta_23_W_22_.

Element	Cr	Mn	Fe	Ta	W
Pauling electronegativity	1.66	1.55	1.83	1.50	2.36

**Table 3:** The contents of each element on the surfaces of HT1, HT2 and HT3, as detected by EDS and XPS.

Samples	Elements	O	Cr	Mn	Fe	Ta	W
		at.%					
HT1	EDS	42.84	23.05	30.42	1.25	0.79	1.66
		-	40.32	53.21	2.19	1.38	2.9
	XPS	66.98	7.9	21.08	1.89	0.62	1.51
HT2	EDS	43.06	23.32	30.75	1.01	0.59	1.26
		-	40.96	54	1.78	1.04	2.22
	XPS	65.98	9.23	20.82	2.72	0.48	0.76
HT3	EDS	42.37	23.92	29.98	1.63	0.56	1.54
		-	41.51	52.02	2.83	0.97	2.67
	XPS	65.8	8.78	20.52	2.76	1.02	1.12

## Data Availability

Not applicable.
